# Protein folding mechanism revealed by single-molecule force spectroscopy experiments

**DOI:** 10.52601/bpr.2021.210024

**Published:** 2021-10-31

**Authors:** Hao Sun, Zilong Guo, Haiyan Hong, Ping Yu, Zhenyong Xue, Hu Chen

**Affiliations:** 1 Research Institute for Biomimetics and Soft Matter, Fujian Provincial Key Lab for Soft Functional Materials Research, Department of Physics, Xiamen University, Xiamen 361005, Fujian, China; 2 Center of Biomedical Physics, Wenzhou Institute, University of Chinese Academy of Sciences, Wenzhou 325000, Zhejiang, China; 3 Oujiang Laboratory, Wenzhou 325000, Zhejiang, China

**Keywords:** Protein folding, Free energy landscape, Force spectroscopy, Molten globule state, Transition state

## Abstract

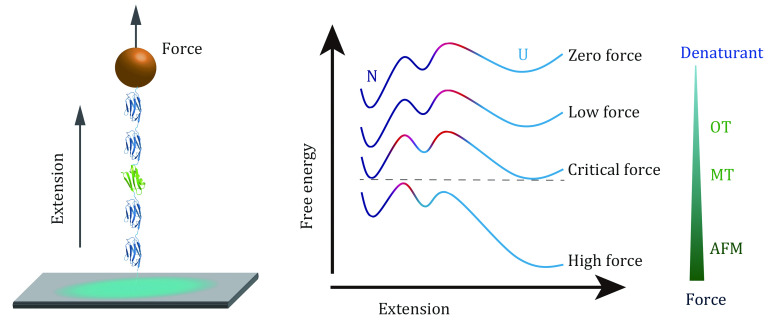

Force spectroscopy experiments use mechanical force as a control factor to regulate the folding and unfolding process of proteins. Atomic force microscopy has been widely used to study the mechanical stability of proteins, and obtained unfolding forces and unfolding distance of different proteins, while recently, more low force folding and unfolding measurements were done by optical tweezers and magnetic tweezers. Due to the relatively small distortion of the free energy landscape, low force measurements give the free energy landscape information over bigger conformational space. In this review, we summarize the results of force spectroscopy experiments on different proteins. The unfolding distance obtained at high forces by atomic force microscopy are mostly smaller than 2 nm, while the unfolding distances at low forces distribute over a larger range: from a negative value to more than 6 nm. The sizes of the transition states at low force are ~4 nm for most compact two-state globular proteins, which indicates that this transition state might be the general free energy barrier separating the unfolded state and the theoretically predicated molten globule state. Up to now, only a limited number of proteins has been studied at low forces. We expect that more and more proteins with different conformations will be studied at low forces to reveal the general protein folding mechanism.

## INTRODUCTION

**Figure 1:**
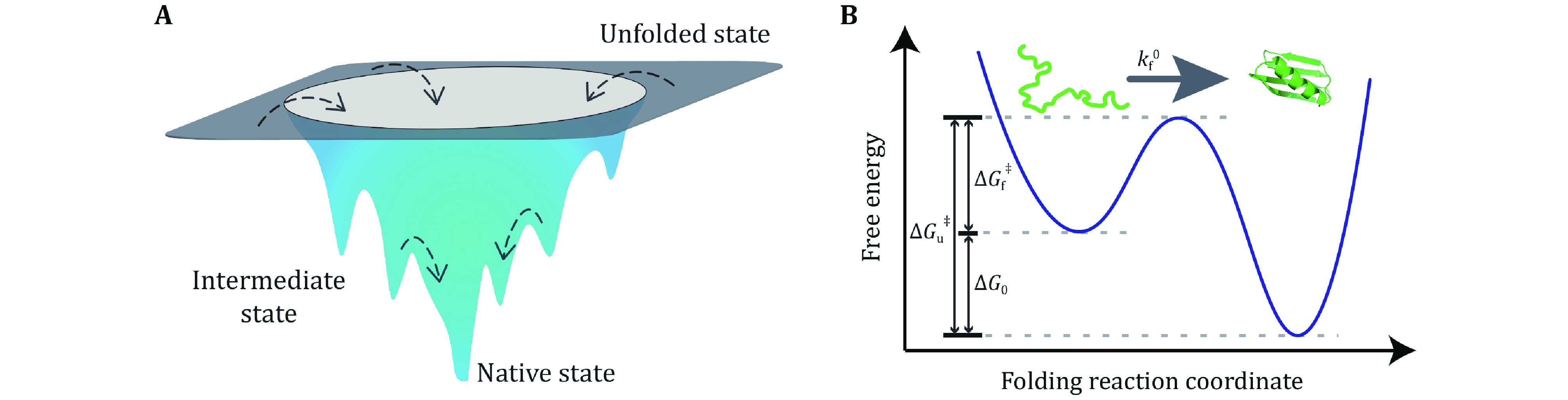
Schematic diagram of free energy landscapes in three-dimensional space and along a single reaction coordinate. **A** Schematic funnel-shaped energy landscape of a protein. The unfolded state of a protein has a large number of microscopic conformations, while the native state has a well-defined single conformation with minimal free energy. Multiple transition states may coexist between the unfolded and folded states (Bryngelson* et al.* 1995; Wolynes* et al.* 1995). **B** Typical free-energy profile of the free energy landscape of a two-state protein. The protein travels from the unfolded state through a transition state to its native state at a folding rate *k*_f_^0^. The free energy difference between the unfolded and folded states, ∆*G*_0_, defines the thermodynamic stability of a protein. The folding barrier ∆*G*_f_^‡^ and the unfolding barrier ∆*G*_u_^‡^ determine the folding rate and unfolding rate, respectively

Protein folding mechanism is an open question in the field of biophysics for more than half century. One-dimensional amino acid sequence information of protein determines the three-dimensional structure of its native state and how fast it folds (Dill* et al.* 2008; Dill and MacCallum 2012; Finkelstein 2018). Native state of a protein is at the global minimal point on the free energy landscape (Fig. 1). Both static native structure and conformation transitions, including folding and unfolding transitions, are important to the function of each protein (Radford 2000).

X-ray refraction, nuclear magnetic resonance (NMR), and electron microscopy techniques have been developed and used to resolve the native structure of proteins. Biochemical methods have been used to trigger the folding and unfolding transitions of proteins to study their folding mechanism (Bartlett and Radford 2009). With the advance of computation capability and development of force field and simulation algorithm, molecular dynamic simulation can fold small proteins in microsecond to millisecond time scale (Bolhuis 2009; Freddolino* et al.* 2010; Best 2012; Piana* et al.* 2014). Based on the available structure of more than 150 thousand proteins, an artificial intelligence algorithm is able to predict the native structure of small proteins with more than 90% accuracy (Senior* et al.* 2020). But the folding mechanisms of proteins, especially proteins which can fold to pathogenic fibrous forms, like prion and Aβ proteins in Mad Cow and Alzheimer’s diseases, are still far from understanding totally (Dobson 2004; van der Kant* et al.* 2020).

In recent years, single-molecule force spectroscopy (SMFS) is increasingly active in the exploration of protein folding, conformational changes, assembly and function (Lipman* et al.* 2003; Hughes and Dougan 2016). To date, atomic force microscopy (AFM), optical tweezers (OT), and magnetic tweezers (MT) are the most widely used techniques in SMFS (Neuman and Nagy 2008). Comparing to biochemical methods using denaturant, free energy landscape tilts by the mechanical force along the force direction in a well-defined manner. Therefore, the force manipulation techniques give more quantitative information of the free energy landscape of proteins which determines both the stability and dynamic process of proteins.

Among AFM, OT, and MT techniques, AFM was firstly used to stretch proteins (Rief* et al.* 1997; Carrion-Vazquez* et al.* 1999). It was found that various proteins have different mechanical stability. At the same pulling speed, some proteins can sustain hundreds of pico-Newtons (pN), such as titin I27 and GB1, while some other proteins will unfold at tens of pN, such as spectrin (Rief* et al.* 1999). It was found that the unfolding force of a protein measured by AFM is related to the pulling geometry of the protein. Proteins can sustain a relatively larger shearing force than the unzipping force (Forman and Clarke 2007).

Due to the mechanical drift of AFM, the stretching cycle must be finished in several seconds. It is challenging to explore the proteins’ response to low forces by AFM, especially the mechanical stable proteins. At a high force, when the force-bearing bond is broken, then a small single domain protein commonly will unfold totally. Even if there are barriers with longer extension, the force will suppress them to make them undetectable.

Recently, OT and MT were used to investigate protein unfolding and folding problems, and force-dependent unfolding and folding dynamics at forces lower than 10 pN can be studied (Cecconi* et al.* 2005; Chen* et al.* 2011, 2013). In OT experiments, two beads coated with different bioconjugation molecules can be manipulated with two independently controlled optical traps (Whitley* et al.* 2018). The complicated folding process of complex multidomain proteins in the presence of chaperones and co-translational folding of a polypeptide just synthesized by ribosome can be studied by optical tweezers (Bustamante* et al.* 2020). The advantage of magnetic tweezers is their large force range, intrinsic force capability, and stability (Chen* et al.* 2011, 2015). The force from zero to more than 150 pN can be achieved easily with permanent rare-earth magnets and 2.8-μm diameter dynabeads (Guo* et al.* 2020). And the same protein tether can be continuously unfolded and refolded for hours to days (Popa* et al.* 2016; Yuan* et al.* 2017).

In this review, we firstly introduce the protein folding problem. After that, a summary of traditional biochemical methods and their typical results is outlined. Then we briefly described the technical principles of the SMFS techniques, including AFM, OT, and MT. The experimental results obtained by AFM at relatively large forces, and the recent results obtained by OT and MT at low forces are summarized. The correlations between protein sizes, mechanical parameters and folding/unfolding rates are analyzed for tens of different proteins. At last, our perspective of the protein folding problem by force spectroscopy methods is given.

## BIOCHEMICAL EXPERIMENT TO STUDY PROTEIN FOLDING

**Figure 2:**
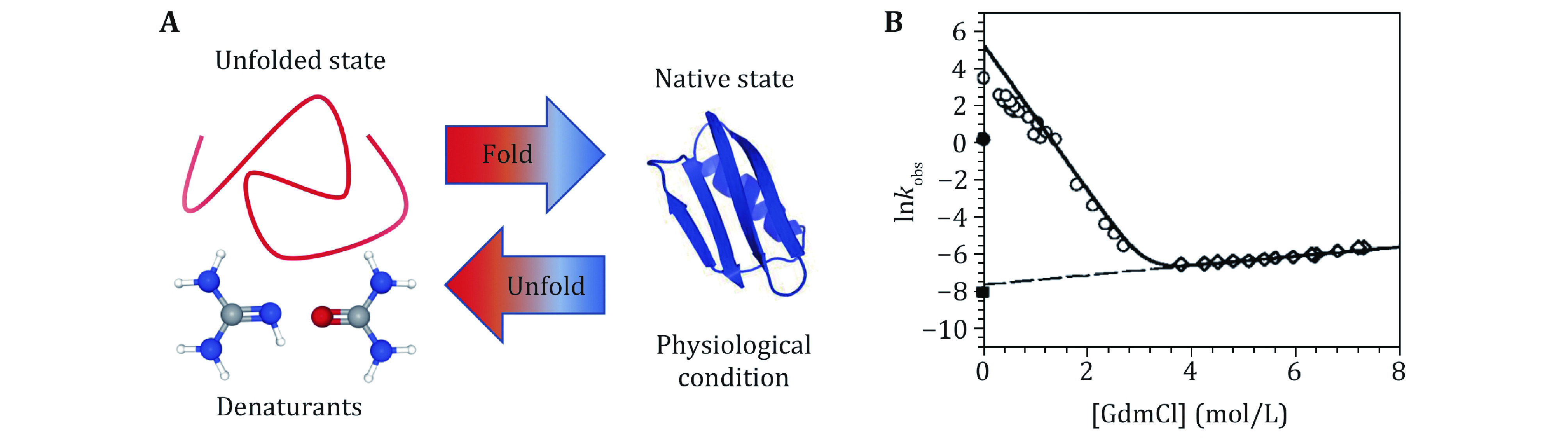
Biochemical technique to study protein folding and unfolding dynamics with denaturants. **A** Sketch of protein denaturation induced by denaturants. Proteins stay at unfolded state in high concentration denaturants solution, and at native state in physiological solution. **B** The typical experimental results of the observed relaxation rate as a function of the concentration of GdmCl. Reprinted with permission from the National Academy of Sciences (Carrion-Vazquez* et al.* 1999)

Different kinds of biochemical techniques have been applied to measure folding and unfolding rates of bulk protein ensemble in solution and provide important information, such as folding free energy, and the free energy difference between different conformations (Hu* et al.* 2016). Most proteins stay at their native state under physiological conditions. Usually, the concentration of denaturant (McCallister* et al.* 2000), temperature (Religa* et al.* 2005) or pH (Balbach* et al.* 1997) of solution can be precisely controlled to induce proteins denaturation. Guanidine Hydrochloride (GmdCl) and urea were widely used as the denaturant in protein folding/unfolding research (Fig. 2). Most proteins can fold and unfold reversibly when changing the concentration of the denaturant, and a stopped-flow instrument can change the denaturant concentration in a few milliseconds (Park* et al.* 1999; Lipman* et al.* 2003). Conformation transition rates can be quantitatively characterized by spectral methods (UV CD, intrinsic tryptophan fluorescence), nuclear magnetic resonance (NMR), or Mass Spectra (MS) methods. A detailed summary of experimental techniques working together with bulk biochemical denaturation experiments can be found in published reviews (Maxwell* et al.* 2005; Bartlett and Radford 2009).

Bulk biochemical experiments measure the average signals from all protein molecules in the detection region. But protein unfolding and folding transitions are stochastic processes, and the conformation transition of each protein molecule happens non-simultaneously. Therefore, bulk experiments easily lose the information from transient intermediate states, which can be overcome by single molecule experiments.

## SINGLE MOLECULE FORCE SPECTROSCOPY TECHNIQUES

In the last three decades, SMFS has gradually become a major technique for investigating protein folding with its distinct advantages. SMFS breaks through the limitations of traditional biochemical techniques and can efficiently analyze the dynamic transitions of proteins under mechanical regulation at the single-molecule level. Popular SMFS techniques include AFM, OT, and MT (Neuman and Nagy 2008).

**Figure 3:**
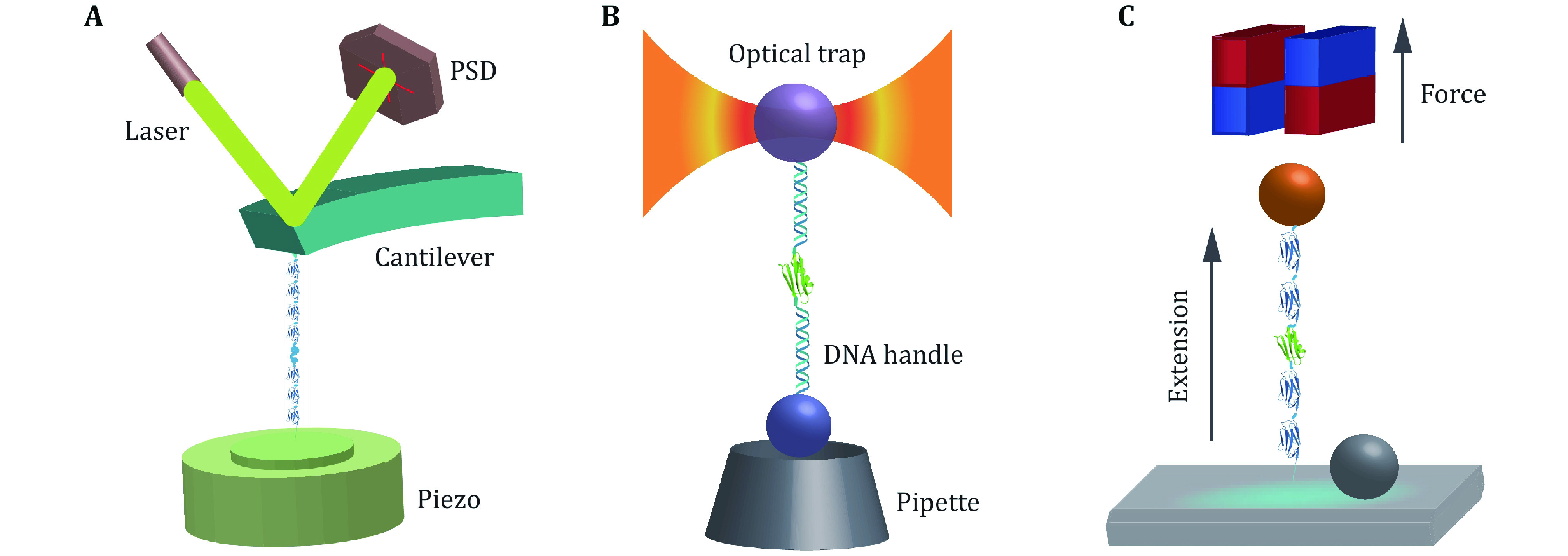
Cartoon schematics of SMFS techniques. **A** AFM: the poly-protein with repeating protein domains is tethered between the cantilever tip and the substrate surface. The retraction of the piezo stage in the axial direction increases the distance between the cantilever and the substrate to stretch the protein, and the force is measured from the deflection of the cantilever. **B** OT: the target protein is linked between a microsphere in the optical trap and another microsphere held by the micropipette via two DNA handles. Controlling the displacement of the microsphere in the optical trap, the force applied to the target protein and the extension changes can be recorded. **C** MT: the protein of interest is attached between a superparamagnetic bead and cover glass surface. A pair of permanent magnets located over the sample chamber imposes a constant force on the protein, while the extension is measured through real time analysis of the microscopic bead images

The principle of the AFM-based SMFS is shown in Fig. 3A. The ends of a constructed biological molecule are attached between the probe and the substrate by specific bioconjugation (Hinterdorfer and Dufrene 2006; Edwards* et al.* 2021) or non-specific binding (Rief* et al.* 1997). The biomolecule of interest is stretched by moving the piezoelectric-controlled micro-cantilever or substrate of AFM, and the resulting force and extension are measured in real time. The advantages of AFM are its high spatial and temporal resolution. The major disadvantage originates from the relatively high stiffness of the cantilever, which leads to a high minimal force and is hard to realize accurate measurements at forces smaller than 10 pN. Note that recently Perkins lab developed an AFM technique to study the force response to forces less than 10 pN for fast folding proteins with the modified AFM cantilever (Edwards* et al.* 2021). Another drawback of AFM is the mechanical drift caused by changes in the relative distance between the substrate and the cantilever, which can accumulate over time leading to severe system drift. Therefore, each stretching cycle by AFM is usually performed in a time scale of several seconds.

OT uses a microscope objective with a high numerical aperture to focus the laser to form a potential well of a microsphere with a diameter of several hundred nanometers to several micrometers (Ashkin* et al.* 1986) (Fig. 3B). When the microsphere moves away from the center of the optical potential well, the trap exerts a recovery force on the microsphere. The microspheres captured by OT are typically attached to the protein of interest through two DNA handles. The precision offered by OT with low-noise, low-drift, and high spatial-temporal resolution (Choudhary* et al.* 2019) is accompanied by non-negligible shortcomings, such as local heating (Peterman* et al.* 2003; Seol* et al.* 2006) and optical damage (Liang* et al.* 1996; Neuman* et al.* 1999) of the trapped specimens.

Different from AFM and OT, an intrinsic constant force is applied on the paramagnetic bead by gradient magnetic field (Fig. 3C). The principle of force generation in MT does not rely on the microscope, its force control and optical measurement are independent, which provides MT the capability to continuously measure the same individual molecule for several hours or even days (Chen* et al.* 2015; Lof* et al.* 2019). In addition, MT can easily twist molecules by rotating magnets, which is of great importance in the study of DNA super-coiling properties (Strick* et al.* 2000; Charvin* et al.* 2005; Gore* et al.* 2006). MT applies an intrinsic constant force to the biomolecule of interest, while AFM and OT need sophisticated feedback systems to maintain constant force roughly (Schlierf* et al.* 2004). Furthermore, the feasible force range of MT is from zero to more than 150 pN (Guo* et al.* 2020), which covers the full range of physiological forces of a single biomolecule in cells.

The single-molecule force spectroscopy provides a powerful method for investigating the transition of molecules along with specific reaction coordinates. In these techniques, molecules and complexes of interest are subjected to pN levels of the applied force by experimental devices through a certain length of the molecular handle, and their response to stretching is recorded. Theoretical efforts have greatly facilitated the link between molecular response and free energy landscape, thus providing sufficient kinetic information. Note that values of dynamics parameters taken out of the context of the experiment setup, the pulling spring stiffness (K), the length of handle (L), and the loading rate are meaningless (Maitra and Arya 2010; Maitra and Arya 2011; Noy 2011). For example, the deviations are negligible (<5%) for small K and large L whereas the deviations can be as high as 35% for the largest K (Maitra and Arya 2011).

## PROTEIN UNFOLDING DYNAMICS AT LARGE FORCES

Based on a vast amount of available publications, we summarize the experimental results of two-state proteins by AFM. The dataset exhibited in Table 1 covers the mechanical unfolding properties of 30 proteins, which includes the protein structure information and some thermodynamic and kinetic characteristics. It is remarkable that certain universal principles are already well accepted. The arrangement of the secondary structure of the protein is of particular importance. Various proteins with distinct topologies have different mechanical properties (Li 2007; Hughes and Dougan 2016). For example, proteins can be roughly ranked according to the content and arrangement of their secondary structures — proteins with structures of all α-helix are relatively mechanically weak, while those containing multiple β-strands have high mechanical stability. The pulling geometry of proteins also plays an important role in the unfolding force, where the force for shearing geometry is greater than that for unzipping geometry.

**Table 1:** The mechanical unfolding characteristics of various protein domains at large forces studied experimentally by AFM

No.	Protein name	PDB ID	a.a. number	Pulling distance (nm)	*x*_u_ (nm)	*F*_u_ (pN) at 400 nm/s	*k*_u_^0^ (s^−1^)	SCOP			Reference
								class	α (%)	β (%)	
1	I27	1TIT	89	4.3	0.25	196	0.00033	all β	-	64	Carrion-Vazquez* et al.* 1999
2	I27	1TIT	89	4.3	0.29	168	0.002	all β	-	64	Brockwell* et al.* 2002; Hoffmann* et al.* 2013
3	Tenascin	1TEN	89	3.1	0.30	137	0.00046	all β	-	59	Oberhauser* et al.* 1998
4	FNIII_13_	1FNH	90	3.3	0.34	85	0.022	all β	-	54	Oberhauser* et al.* 2002
5	FNIII_10_	1FNF	93	3.4	0.38	75	0.02	all β	-	53	Oberhauser* et al.* 2002
6	FNIII_1_	1OWW	93	3.5	0.17	215	0.004	all β	-	53	Oberhauser* et al.* 2002
7	*Tm* Csp	1G6P	65	1.3	0.49	78	0.015	all β	-	43	Hoffmann* et al.* 2013
8	FNIII_2_	2KBG	102	3.9	0.17	215	0.004	all β	-	39	Oberhauser* et al.* 2002
9	I1	1G1C	97	5.2	0.35	120	0.005	α + β	5	64	Li and Fernandez 2003
10	GB1	1PGA	56	2.6	0.17	178	0.039	α + β	25	62	Cao* et al.* 2006
11	NuG2	1MI0	56	2.6	0.42	60	0.04	α + β	25	62	He* et al.* 2015
12	protein L	1HZ6	67	3.2	0.22	136	0.05	α + β	23	53	Brockwell* et al.* 2005
13	C2A	2R83	124	2.8	0.72	50	N/A	α + β	6	47	Fuson* et al.* 2009
14	Zn-pfRD	1ZRP	53	0.6	0.14	172	0.1	α + β	22	43	Zheng and Li 2011
15	C2B	1TJX	159	2.3	0.41	100	N/A	α + β	22	42	Fuson* et al.* 2009
16	Top 7	1QYS	93	3.0	0.21	180	0.06	α + β	38	40	Sharma* et al.* 2007
17	AcP	1APS	98	2.5	0.6	50	0.03	α + β	23	36	Arad-Haase* et al.* 2010
18	SKβ	1C4P	136	5.1	0.13	220	0.06	α + β	11	35	He and Li 2017
19	PAS-B	1X0O	119	2.4	2	32	0.00003	α + β	24	33	Gao* et al.* 2012
20	Fe(III)-pfRD	1CAD	53	1.1	0.11	227	0.15	α + β	30	32	Zheng* et al.* 2013)
21	Ubiquitin	1UBQ	76	3.8	0.23	220	0.00005	α + β	29	31	Chyan* et al.* 2004
22	Ubiquitin	1UBQ	76	3.8	0.14	150	0.0375	α + β	29	31	Schlierf* et al.* 2004
23	Ubiquitin	1D3Z	76	3.8	0.25	220	0.0004	α + β	29	31	Carrion-Vazquez* et al.* 2003
24	Ubiquitin (48-C)	1D3Z	76	2.7	0.63	90	0.0004	α + β	29	31	Carrion-Vazquez* et al.* 2003
25	SAK	1SSN	136	1.0	0.41	68	0.05	α + β	12	30	He and Li 2017
26	AFV3-109	2J6B	109	1.2	0.24	113	1.8	α + β	31	27	He* et al.* 2012
27	DHFR-MTX	1RG7	159	1.8	0.37	80	N/A	α + β	22	26	Ainavarapu* et al.* 2005
28	Apo-G6	3FFN	116	3.5	1.73	24	0.09	α + β	33	21	Lv* et al.* 2014
29	Holo-G6	1P8X	116	3.5	1.6	40	0.00005	α + β	33	21	Lv* et al.* 2014
30	Barnase	1BNR	110	2.6	0.58	55	0.00023	α + β	20	17	Best* et al.* 2001
31	T4 lysozyme	1L63	164	0.8	0.75	50	0.055	α + β	66	16	Peng and Li 2008
32	cHiPIP	2FLA	83	3.0	0.15	165	0.38	α + β	33	15	Li and Li 2018
33	SptP	1G4U	383	6.5	1.5	21	0.2	α + β	56	15	Leblanc* et al.* 2021
34	Cam DomC	1CFC	70	1.8	2	15	0.02	α + β	58	8	Junker* et al.* 2009
35	SopE2	1R9K	172	3.4	1.4	14	0.7	α + β	79	2	Leblanc* et al.* 2021
36	GA	2FS1	56	4.1	1.5	20	0.39	all α	80	-	Xiao and Li 2019
37	spectrin	1AJ3	110	4.2	1.7	30	0.00003	all α	89	-	Rief* et al.* 1999
Values for the unfolding force at 400 nm/s have been interpolated when necessary. Pulling distance is the distance between stretching points in native structure. The values of *x*_u_ were mostly obtained by fitting the dependence of unfolding force at different pulling speeds using the Bell model, and individually derived from Monte Carlo simulations. *k*_u_^0^ is the intrinsic unfolding rate at zero force; SCOP is an abbreviation for Structural Classification of Proteins; “N/A” represents not available											

**Figure 4:**
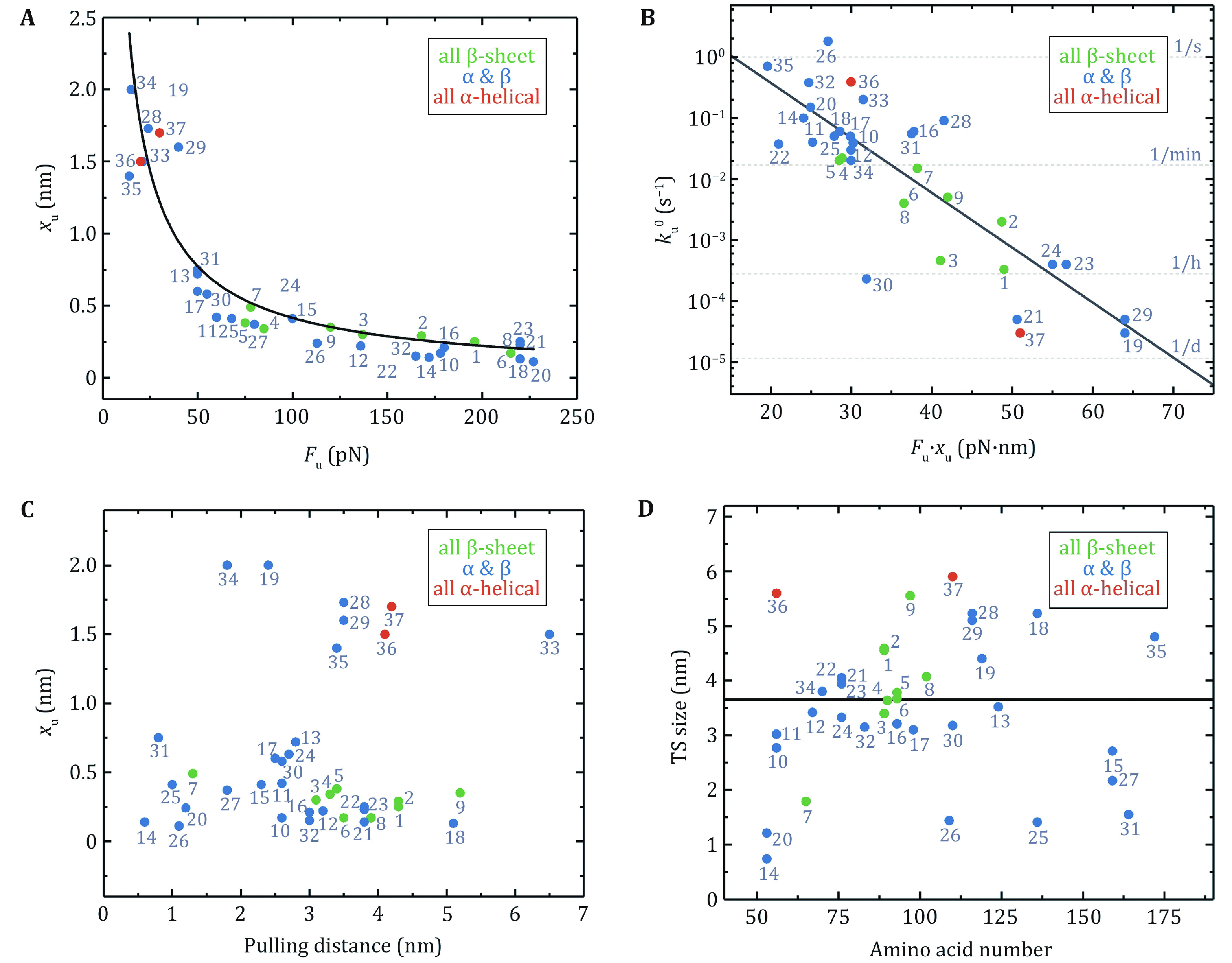
The relationships of mechanical properties of proteins in Table 1 obtained by AFM. Protein numbering in all pictures is the same as in Table 1. **A** The relationship between unfolding distance (*x*_u_) and mean unfolding force (*F*_u_) at a pulling speed of 400 nm/s. The data can be described by the Bell–Evans model (black solid line) for the most probable unfolding force *F*_u_ = (*k*_B_T/*x*_u_)ln[*r* · *x*_u_/(*k*_u_^0^ · *k*_B_T)] using a fixed loading rate (*r* = 200 pN/s) and unfolding rate at zero force (*k*_u_^0^ = 0.2 s^−1^) (Leblanc* et al.* 2021). **B** The dependence of the unfolding rate at zero force (*k*_u_^0^) on the product of the unfolding force and the unfolding distance (*F*_u_ · *x*_u_). Proteins with a high value of *F*_u_ · *x*_u_ unfold several orders of magnitude more slowly than proteins with a low value of *F*_u_ · *x*_u_. The linear fitting has *R*^2^ of 0.70. **C** *x*_u_ is plotted against the pulling distance. **D** The transition state size is plotted as a function of the number of amino acids in each protein. The black solid line is the average transition state size: 3.7 ± 1.5 nm (mean ± SD)

The previous review articles provided an understanding of the relationship between the native structures of proteins and their mechanical stability and malleability. A crucial structural region in a protein that is of responsibility for the resistance to stretching is often composed of hydrogen bonds among adjacent β-strands (Hoffmann* et al.* 2013; Hughes and Dougan 2016). As a result, this feature grants mechanical stability to proteins, and broken of those hydrogen bonds acts as a rate-limiting step for unfolding at large force. Figure 4A reveals a strong correlation between the unfolding force (*F*_u_) and the unfolding distance (*x*_u_), which is the distance from the native state to the unfolding transition state. Proteins with low *F*_u_ possess a large *x*_u_ and proteins with high *F*_u_ own a small *x*_u_. The global tendency, meanwhile, is that pure α-helix proteins are mechanically weaker than proteins with mixed α-helix/β-sheet and all β-sheet proteins. LeBlanc *et al*. have successfully fitted the shape of the *x*_u_ vs. *F*_u_ plot by using the Bell-Evans equation *F*_u_ = *k*_B_T/*x*_u_ ln[*r* · *x*_u_/(*k*_u_^0^ · *k*_B_T)] with loading rate *r* = 200 pN/s and zero-force unfolding rate *k*_u_^0^ = 0.2 s^−1^, which highlights the role of *x*_u_ in governing the mechanical stability of proteins with different structures (Leblanc* et al.* 2021).

Clearly, the presence of external forces tilts the energy landscape of the protein, which consequently lowers the unfolding energy barrier. Here, the product of *F*_u_ and *x*_u_ is crucial, which mirrors the work done by the external force over the distance from the native state to the transition state of the protein. It can be, hence, an indicator of the change in the unfolding free energy barrier. Figure 4B exhibits the dependence of the zero-force unfolding rate *k*_u_^0^ on the product of *F*_u_ and *x*_u_. Obviously, *F*_u_ · *x*_u_ has a strong correlation with *k*_u_^0^. Proteins with lower *F*_u_ · *x*_u_ unfold faster than those with higher *F*_u_ · *x*_u_. Hoffmann *et al*. proposed that a lower unfolding barrier for proteins with lower *F*_u_ · *x*_u_ could explain this observation (Hoffmann* et al*. 2013).

The Bell model usually gives a nice description of the force-dependent unfolding rates over the AFM force range for two-state proteins. Unfolding distance, *x*_u_, as an important parameter determined by the model, is a perfect indicator of protein deformation when it crosses the potential barrier. *x*_u_ indicates the level of protein softening. The larger the *x*_u_, the more significant the protein malleability, and vice versa. Figure 4C shows *x*_u_ as a function of the distance between pulling points in the native state (defined as pulling distance), which demonstrates little correlation. But one can easily notice that *x*_u_ of proteins consisting of all β-sheet chains is rather small, and *x*_u_ of proteins with pure α-helix chains is slightly larger, while proteins with mixed α-helix and β-strands have *x*_u_ over a large range. The inter-strand hydrogen bonds between β-strands provide more mechanical stability than hydrophobic contacts between α-helixes.

The size of the unfolding transition state can be calculated by the sum of the distance between pulling points in the native state and *x*_u_ of the protein. The number of amino acids in a protein determines the size of the globular native state. Nevertheless, there is no apparent correlation between the size of the transition state obtained at large force by AFM and the amino acid numbers of the proteins (Fig. 4D).

## PROTEIN FOLDING AND UNFOLDING DYNAMICS AT LOW FORCES

**Figure 5:**
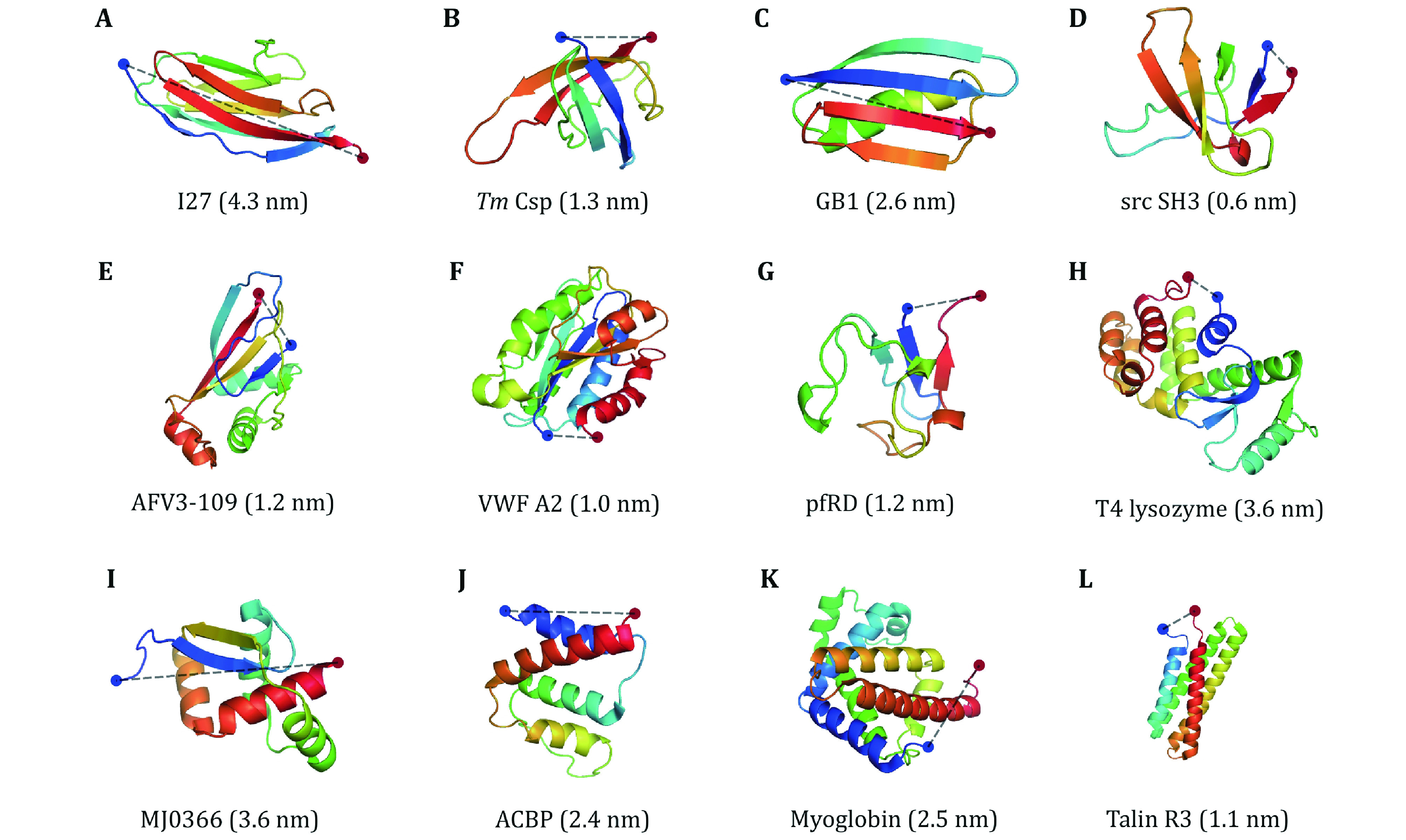
Three-dimensional cartoon representations of the native structure of all proteins studied by OT and MT (Table 2). The blue and red dots indicate the N- and C-termini of the protein, respectively. The N–C distances are given in parentheses. NuG2 is a computationally designed variant of the protein GB1, whose structure is not shown here

Apart from elucidating the mechanical properties of proteins under large forces by AFM (Table 1), recently, the force-dependent folding and unfolding rates of some proteins are studied by OT and MT at low forces adjacent to the critical force at which the protein has 50% probability in the unfolded state. For a two-state protein, the protein transits between the unfolded state and the native state by crossing the transition state. From the force-dependent folding rate, the size of the folding transition state can be determined, which is usually identical to the size of the unfolding transition state at the same force range (Su* et al.* 2021), indicating the folding and unfolding transitions are through the same pathway. Here we summarize the properties of proteins whose force response has been measured at low forces by OT and MT in recent years (Fig. 5 and Table 2), and performed some correlation analysis.

**Table 2:** The mechanical folding and unfolding characteristics of various protein domains at low forces studied experimentally by OT and MT

No.	Protein name	PDB ID	a.a. number	Pulling distance (nm)	*x*_u_ (nm)	Force range (pN)		*k*_u_^0^ (s^−1^)	*k*_f_^0^ (s^−1^)	∆*G*_0_ (*k*_B_T)	SCOP			Reference
						Min force	Max force				Class	α (%)	β (%)	
1	I27	1TIT	89	4.3	−0.33	4.5	20	0.00034	2.1	8.3	all β		62	Yuan* et al.* 2017
2	*Tm* Csp	1G6P	66	1.3	2.9	5	7	0.00042	400	12.2	all β		42	Unpublished work
3	GB1	1PGA	56	2.6	1	5	9	0.0004	150	11.9	α + β	25	62	Guo* et al.* 2020
4	NuG2	1MI0	56	2.6	0.61	10	40	0.0018	24000	16.7	α + β	25	62	Lei* et al.* 2017
5	src SH3	1SRL	56	0.6	2.1	4	11	0.027	25	6.8	α + β	5	42	Su* et al.* 2021
6	AFV3-109	2J6B	109	1.2	3.1	5	12	0.003	180	12.3	α + β	31	27	He* et al.* 2019
7	VWF A2	3GXB	177	1.0	2.45	7	16	0.000036	5.1	11.9	α + β	43	22	Lof* et al.* 2019
8	pfRD(L)	1BRF	53	1.2	2.31	4	8	0.14	16000	11.6	α + β	18	21	Li and Li 2020
9	pfRD(H)	1BRF	53	1.2	3.76	9	12	0.000076	15000	19.1	α + β	18	21	Li and Li 2020
10	WT T4 Lysozyme (16-159)	1L63	162	3.6	0.64	20	60	0.0014	N/A	24.05	α + β	67	17	Shank* et al.* 2010
11	MJ0366	2EFV	82	3.6	1.8	12	24	0.0004	80000	21.4	α + β	63	15	Rivera* et al.* 2020
12	Holo-ACBP	1NTI	86	2.4	5.9	8	14	0.0000007	3000	22.18	all α	71	−	Sonar* et al.* 2020
13	Apo-ACBP	1NTI	86	2.4	5.4	8	12	0.00005	4000	18.2	all α	71	−	Sonar* et al.* 2020
14	Myoglobin	1BZ6	153	2.5	1.2	5	16	0.07	800000000	23.16	all α	79	−	Elms* et al.* 2012
15	Myoglobin (53-C)	1BZ6	153	3.9	1	7	18	0.1	15000000	18.83	all α	79	−	Elms* et al.* 2012
16	Talin R3	2L7A	125	1.1	6.49	4	6	0.0025	120000	17.7	all α	86	−	Tapia-Rojo* et al.* 2020
17	Talin R3 IVVI	2L7A	125	1.1	4.27	8	11	0.0000046	1800000	26.7	all α	86	−	Tapia-Rojo* et al.* 2020
The values of *x*_u_ were obtained by fitting the dependence of unfolding rate at successively low forces using the Bell model. *k*_u_^0^ and *k*_f_^0^ are the intrinsic unfolding and folding rates at zero force. “N/A” represents not available														

**Figure 6:**
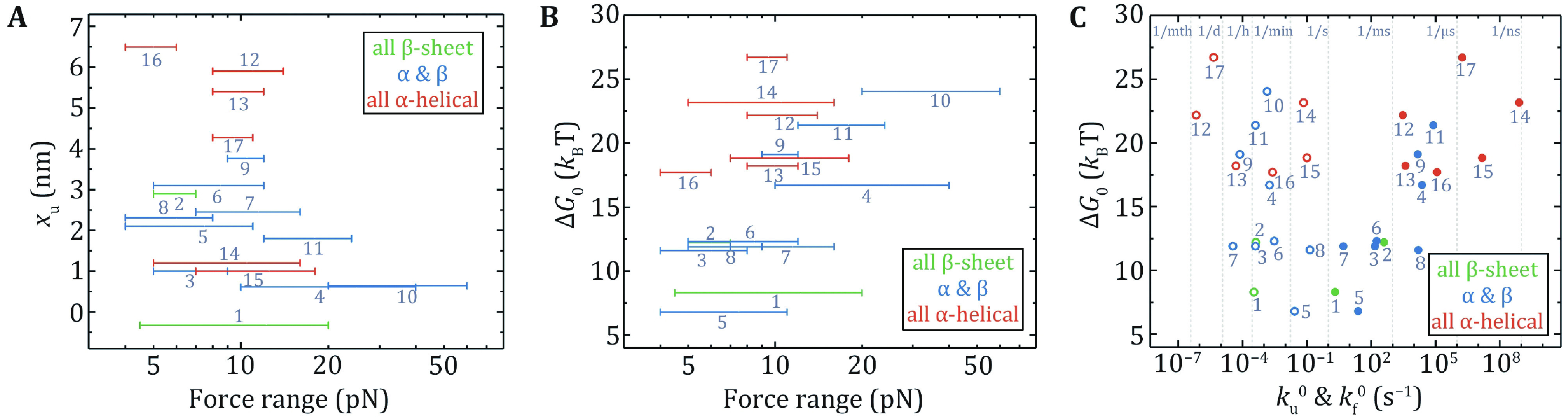
The relationships of mechanical properties of proteins in Table 2 obtained by OT and MT. Unfolding distance *x*_u_ (**A**) and folding free energy Δ*G*_0_ (**B**) are plotted as a function of the experimental force range. **C** Correlation between Δ*G*_0_ and zero-force folding rates (solid circles), and correlation between Δ*G*_0_ and zero-force unfolding rates (open circles). Protein numbering in all the figures is the same as in Table 2

Figure 6A shows *x*_u_ vs. the force range of the measurement. Except for I27 and T4 lysozyme, all proteins had *x*_u_ from 1.0 nm to 6.5 nm, which means that they need to deform significantly to arrive at the transition state in the low force region. The *x*_u_ of I27 is –0.33 nm at forces less than 20 pN, which means that the increasing force actually slows down the unfolding transition (a catch-bond behavior) (Yuan* et al.* 2017). T4 lysozyme only requires a small deformation to unfold mainly due to the coupling effect between its domains (Shank* et al.* 2010). It is noteworthy that NuG2 is a computationally designed fast-folding variant of the protein GB1 (Nauli* et al.* 2001, 2002). NuG2 exhibits different *x*_u_ in distinct force ranges from the data obtained using AFM and OT (He* et al.* 2015; Lei* et al.* 2017). On one side is due to the limitations of different research equipment and on the other side, it confirms that the *x*_u_ of the protein is force-dependent.

The thermodynamic stability of a protein is given by the free energy difference between the unfolded state and the native state. The force-dependent folding free energy ∆*G*(f) can be determined by the ratio of the folding rate *k*_f_(f) and the unfolding rate *k*_u_(f) at low forces: ∆*G*(f) = *k*_B_T · ln(*k*_f_(f)/*k*_u_(f)), from which the zero-force folding free energy ∆*G*_0_ can be obtained. Figure 6B presents the relationship between the experimental force range and ∆*G*_0_. The trend is that as the experimental force increases, ∆*G*_0_ gets larger. A point worth noting is that ∆*G*_0_ of the proteins with all α-helix is larger than most of the proteins containing β-strands. Another finding is that there is a positive correlation between *k*_u_^0^ and ∆*G*_0_, and a negative correlation between *k*_f_^0^ and ∆*G*_0_ (Fig. 6C).

**Figure 7:**
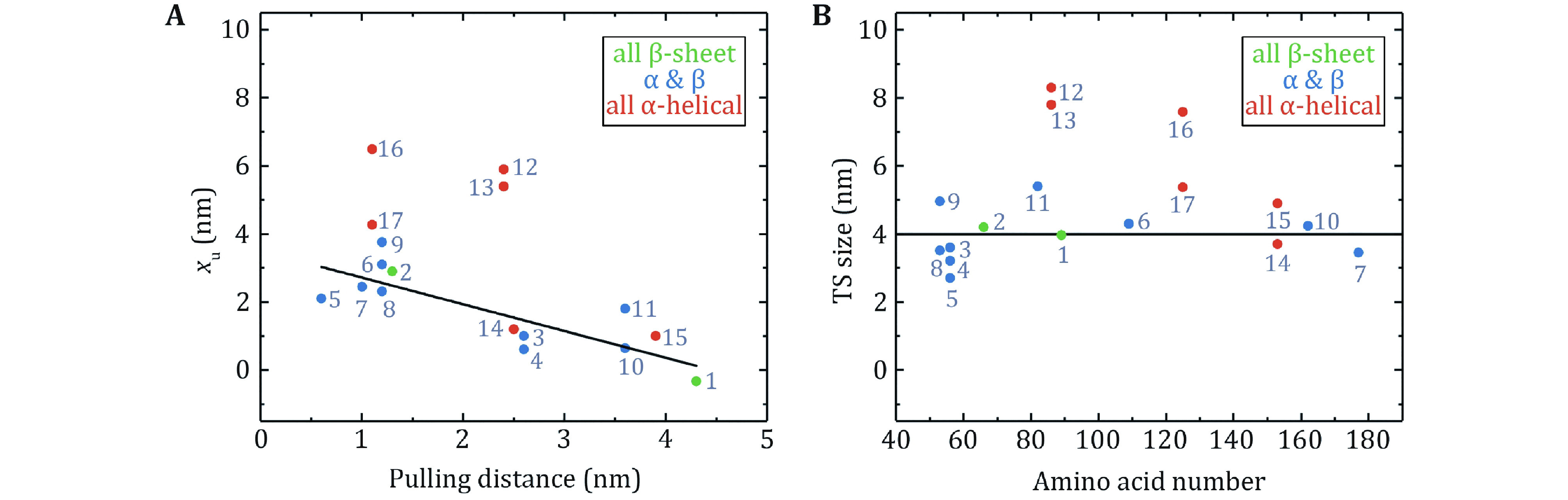
Unfolding distances and size of transition states obtained at low force by OT and MT. **A** The unfolding distance (*x*_u_) plotted as a function of the pulling distance. The linear fitting for proteins with β-strands has an *R*^2^ = 0.68. **B** The transition state size for proteins with different amino acid numbers. The black solid line is the average transition state size of proteins with β-strands: 4.0 ± 0.8 nm (mean ± SD). Protein numbering in both figures is the same as in Table 2

It is notable that *x*_u_ for globular proteins with β-strands obtained from low forces measurement is correlated with the pulling distance (Fig. 7A). The unfolding distance *x*_u_ of proteins decreases as the pulling distance increases, even to a negative value for titin I27 with a pulling distance of 4.3 nm (Yuan* et al.* 2017). For proteins with the N- and C- termini as the pulling points, *x*_u_ of proteins with "shearing" stretching geometry is relatively small because of their large N–C distance, while *x*_u_ of proteins with "unzipping" pulling geometry is generally big due to their small N–C distance.

More interestingly, most proteins display certain uniformity in the size of transition states, which is 4.0 ± 0.8 nm (mean ± SD) (Fig. 7B). It is perhaps of pivotal implication in the protein folding mechanism, which will be discussed in the next section. On the other hand, for most proteins composed of only α-helix secondary structure, such as α-helix bundles, the hydrophobic core formed by inter-helix interactions as the predominant potential barrier demands a relatively large deformation to overcome the barrier during the unfolding process.

## SUMMARY AND PERSPECTIVES

In conclusion, we have analyzed the correlations of major parameters of proteins, including the unfolding force (*F*_u_), the transition distance (*x*_u_), the folding and unfolding rates (*k*_f_^0^ and *k*_u_^0^) at zero force, and the folding free energy (∆*G*_0_), investigated by SMFS at different force ranges. Although these correlations stand for the average behavior of numerous different proteins, it remains a helpful observation to predict the mechanical stability of the proteins of interest and to judge the degree of deviation of the studied proteins from the average behavior.

**Figure 8:**
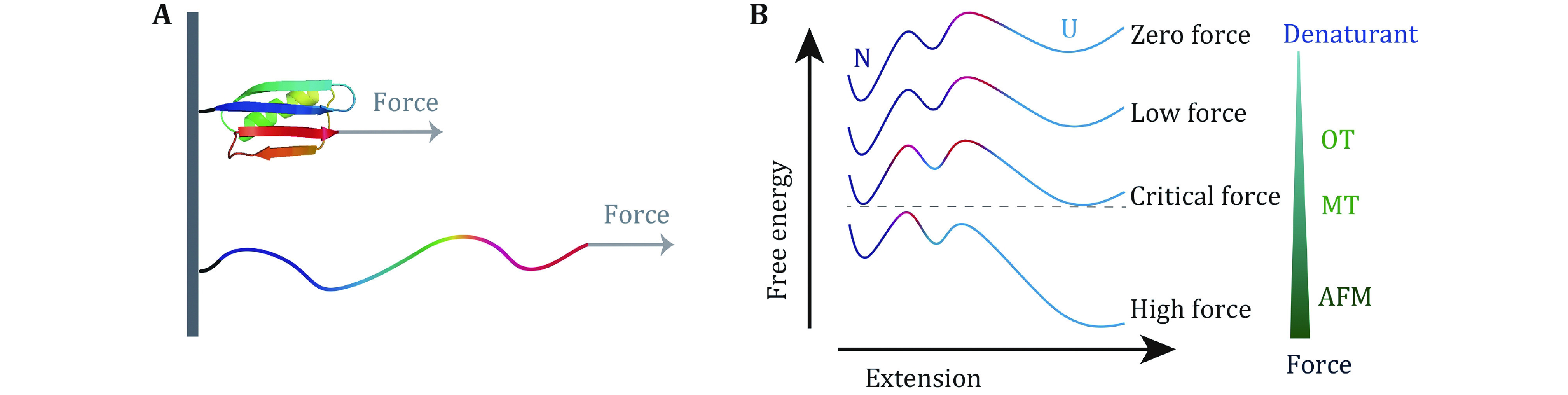
Detailed protein free energy landscape revealed by SMFS measurement over large force range. **A** Sketch of protein unfolding under stretching force. **B** Free energy landscape with two barriers and an intermediate at different forces. Red color part shows the dominant barrier with the highest free energy. At high force, only the barrier close to the native state can be detected, while at low force, additional information of the free energy landscape over larger conformational space can be studied

In force spectroscopy experiment on protein unfolding studies by AFM, the high force measurement reveals the mechanical stability of proteins which is mainly determined by the strength of a so-called “mechanical clamp” in many proteins. A mechanical clamp is a structural domain of a protein that is responsible for the resistance to stretching. Therefore, this factor grants mechanical robustness of the protein, and breaking of the “mechanical clamp” serves as the rate-limiting step for the unfolding of a protein (Sikora* et al.* 2009, 2010; Hughes and Dougan 2016). From the view point of the free energy landscape, only the local region close to the native state is responsible for the unfolding process in high force measurements (Fig. 8).

To unveil the protein folding mechanism, denaturants and the stretching force are used to trigger the folding and unfolding transitions (Banachewicz* et al.* 2011; Lv* et al.* 2012). Extrapolation to the condition without denaturants or stretching force is usually done to study the folding mechanism at physiological conditions (Banachewicz* et al.* 2011; Nasreen* et al.* 2020; Su* et al.* 2021). If the experimental condition is too far away from the physiological condition, the extrapolation might have problems as the slope of the fitting line might change when the environment condition is approaching the physiological condition (Chen* et al.* 2015; Guo* et al.* 2020; Yuan* et al.* 2017). Therefore, low force measurement provides indispensable information on the protein folding mechanism.

Critical forces at which the protein has an equal folding rate and the unfolding rate is usually smaller than 10 pN. The extrapolation will not deviate too much when it is done based on force-dependent folding rate and the unfolding rate at forces less than 10 pN. We found that sizes of the transition states at low force region are about 4 nm for compact two-state globular proteins. This general transition state is almost independent of the specific structure of each protein. If the unfolded state can be modeled as a random coil, the size of the unfolded state is more than 6 nm for proteins longer than 60 amino acids. Theoretically, a collapsed molten globule state is predicted to form before folding to the native state (Elms* et al.* 2012; Kuwajima 2020). We propose that this 4-nm transition state is the universal barrier between the unfolded state and the intermediate molten globule state (Fig. 8).

Comparing to the large number of available unfolding measurements done by AFM at a high force range, the low force responses of proteins have only been done for a limited number of proteins. To reveal the general protein folding mechanism of different kinds of proteins, from the simple down-hill or two-state single domain proteins to larger proteins with several folding intermediate states, from compact globular proteins to alpha-helix bundle proteins, membrane proteins, and fibrous proteins, from soluble proteins to protein aggregates, we expect that force-dependent folding and unfolding transitions at low forces for a growing number of proteins will be studied in the future.

## Conflict of interest

Hao Sun, Zilong Guo, Haiyan Hong, Ping Yu, Zhenyong Xue and Hu Chen declare that they have no conflict of interest.
